# It’s all in the numbers: Cohesin stoichiometry

**DOI:** 10.3389/fmolb.2022.1010894

**Published:** 2022-10-18

**Authors:** Avi Matityahu, Itay Onn

**Affiliations:** The Azrieli Faculty of Medicine, Bar-Ilan University, Ramat Gan, Safed, Israel

**Keywords:** cohesin, Smc proteins, sister chromatid cohesion, cohesin dimers/oligomers, loop extrusion

## Abstract

Cohesin, a structural maintenance of chromosome (SMC) complex, organizes chromatin into three-dimensional structures by threading chromatin into loops and stabilizing long-range chromatin interactions. Four subunits in a 1:1:1:1 ratio compose the cohesin core, which is regulated by auxiliary factors that interact with or modify the core subunits. An ongoing debate about cohesin’s mechanism of action regards its stoichiometry. Namely, is cohesin activity mediated by a single complex or cooperation between several complexes that organize into dimers or oligomers? Several investigations that used various experimental approaches have tried to resolve this dispute. Some have convincingly demonstrated that the cohesin monomer is the active unit. However, others have revealed the formation of cohesin dimers and higher-order clusters on and off chromosomes. Elucidating the biological function of cohesin clusters and determining what regulates their formation are just two of the many new questions raised by these findings. We briefly review the history of the argument about cohesin stoichiometry and the central evidence for cohesin activity as a monomer vs. an oligomer. Finally, we discuss the possible biological significance of cohesin oligomerization and present open questions that remain to be answered.

## Introduction

Cohesin, a structural maintenance of chromosome (SMC) complex, plays a central role in shaping the three-dimensional structure of the genome. Cohesin controls the 3D landscape of chromatin in the cell nucleus during interphase by extruding chromatin into loops and tethering remote regions of chromatin either in *cis* or *trans.* Interphase chromatin extrusion by cohesin shapes it into loops and topologically associated domains (TADs). After their formation, cohesin stabilizes these structures by *cis* tethering the chromatin at their bases (Kakui and Uhlmann, 2018; Kim and Yu, 2020; Davidson and Peters, 2021; Oldenkamp and Rowland, 2022). Loops and TADs have been implicated in regulating fundamental DNA metabolism processes, including transcription, replication, and repair (Miura and Hiratani, 2022). The second and well-studied function of cohesin is associated with the tethering of sister chromatids in *trans*, a process also known as sister chromatid cohesion (Tanaka et al., 2000; Onn et al., 2008). After DNA replication, the identical DNA molecule products are assembled into distinct chromatin fibers, called sister chromatids, that remain attached until their separation during mitosis. This cohesin-mediated tethering of the sister chromatids ensures their bipolar attachment to the spindle and the fidelity of their segregation in mitosis. The mechanism governing *cis*- and *trans*-activation of cohesion has been the focus of many studies offering elucidation on the structure of cohesin, its conformation changes, and molecular mechanism [for example (Orgil et al., 2016; Higashi et al., 2020; Shi et al., 2020; Pathania et al., 2021; Petela et al., 2021)]. However, much has yet to be learned.

One of the intriguing questions regarding cohesin appeared not long after its discovery. The subject of a still unresolved debate concerns the stoichiometry of the cohesin complex. It is accepted that cohesin is a four-subunit complex (Michaelis et al., 1997; Losada et al., 1998; Tanaka et al., 2000; Haering et al., 2002) and that the ratio among all cohesin subunits in cells is 1:1:1:1 (Ding et al., 2011; Holzmann et al., 2011). However, this result does not reveal how many cohesin monomers are needed to form a functional unit (Huang et al., 2005). In other words, are cohesin functions, chromatin extrusion, and chromatin tethering mediated by a single complex, or does it require cooperation between two or more individual cohesin complexes? Here, we review the arguments that have been presented on both sides, including recent studies and current knowledge on the subject. What can be said in brief is that the answer to this question, as with so many other biological puzzles, is complicated.

## Cohesin structure and the loop extrusion mechanism

**FIGURE 1:**
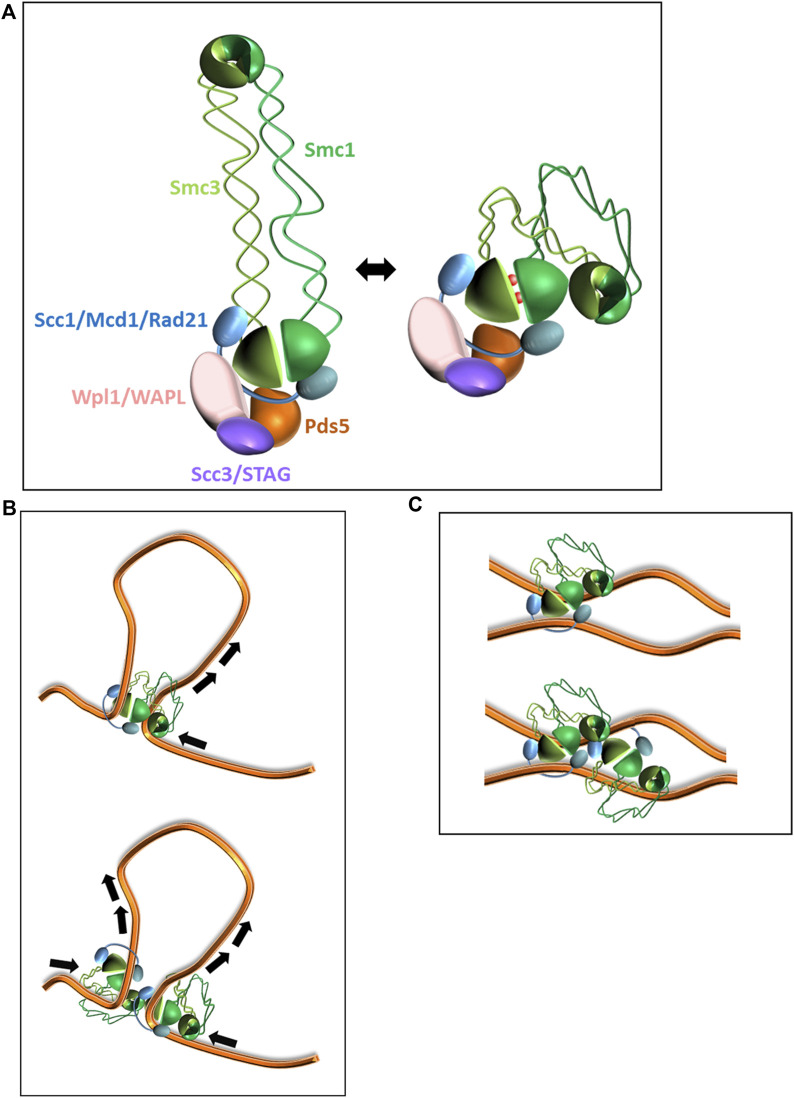
Models of cohesin activity. **(A)**. Architecture of cohesin holocomplex. Subunits’ names in yeast and human are indicated. Open and folded (head-hinge interaction) conformations of the complex are shown. **(B)**. Loop extrusion by cohesin. Shown, a unidirectional extrusion by a monomer (up) or a bidirectional extrusion by a dimer (down). **(C)**. Sister chromatid cohesion. Tethering by a cohesin monomer (up) or a cluster (down) is shown.

Cohesin is composed of Smc1, Smc3, kleisin (Scc1/Mcd1 in yeast, Rad21 in mammalian cells), and Scc3 (STAG in mammalian cells) (Figure 1A) (Losada et al., 1998; Tanaka et al., 2000; Haering et al., 2002). The SMC proteins contain two globular domains called *hinge* and *head*, which are connected by an extended coiled-coil region with an overall structure that resembles a flexible rod. From hinge to head, the length of the SMC protein is about 50 nm Smc1 and Smc3 dimerize through their hinges to form a V-shaped structure. The kleisin interacts with Smc1 and Smc3 heads, restricts their free movement, and reshapes the tripartite structure into a ring. Scc1 also serves as an interaction hub with the fourth core subunit, Scc3, and a set of regulatory subunits: Scc2 (NIPBL in mammalian cells), Pds5, and Wpl1 (WAPL in mammalian cells) (Michaelis et al., 1997; Losada et al., 1998; Tanaka et al., 2000; Onn et al., 2008; Matityahu and Onn, 2018; Matityahu and Onn, 2021). The SMC heads contain two incomplete ATP binding cassette (ABC)-type ATPase domains. The binding of ATP molecules to the half-ATPase sites in Smc1 and Smc3 induces their engagement and the formation of two active ATPases. ATP hydrolysis is stimulated by DNA and causes a conformational change in cohesin, leading to a foldback of the hinge onto the heads (Figure 1A) (Kim and Yu, 2020). DNA is bound between Scc1 and the Smc heads and has another binding site at the hinge domain (Soh et al., 2015; Xu et al., 2018; Shi et al., 2020; Bauer et al., 2021). The conformational changes and dual DNA binding sites are essential elements of cohesin molecular activity (Arumugam et al., 2006; Camdere et al., 2018).

The mechanism by which cohesin organizes chromatin in interphase is called loop extrusion (recently reviewed in (Oldenkamp and Rowland, 2022)). Briefly, repeated cycles of ATP hydrolysis followed by cohesin conformational changes described above result in DNA threading through the SMC lumen at a rate of about 0.5–2 kb/s (Kim et al., 2019; Golfier et al., 2020; Bauer et al., 2021) (Figure 1B). The product of these extractions are chromatin loops of 0.1–1 Mb (average size that varies between organisms). After formation, the extruded loops are organized into TADs by the formation of intra-loop interactions. The 3D organization of chromatin in interphase controls DNA processes such as transcription by defining promoter-enhancer interactions and DNA replication timing through the spatial organization of the replication origins (Kakui and Uhlmann, 2018; Davidson and Peters, 2021; Oldenkamp and Rowland, 2022). TADs have also been suggested to be a functional unit for double-strand DNA break repair as cohesin-mediated loop extrusion drives yH2AX spreading and domain establishment (Arnould et al., 2021). *In vitro* single-molecule experiments showed bi-directional extrusion of DNA by cohesin. However, alternative mechanisms have been suggested to explain this observation (Figure 1B). The most straightforward mechanism is that cohesin monomers extrude DNA bi-directionally (Kim et al., 2019; Bauer et al., 2021). However, this explanation suffers from two weaknesses. First, the cohesin-related SMC complex condensin extrudes DNA unidirectionally, but loop growth velocities mediated by cohesin and condensin are similar. Second, the translocation rate of a cohesin monomer on DNA is approximately half the rate of loop formation. These discrepancies can be explained if extrusion is mediated by a cohesin dimer (Davidson et al., 2019; Golfier et al., 2020).

In contrast to active extrusion, the competing diffusion capture model offers an explanation of extrusion that relies on simple biophysical principles rather than the complex binding patterns of cohesin (Lawrimore et al., 2017; He et al., 2020; Gerguri et al., 2021). Specifically, the model suggests that DNA extrusion results from cohesin motor activity on a floppy chromatin substrate. Cohesin embraces two DNA regions that come into proximity *via* Brownian motion and serves as a stabilizing factor for the loop. Repeated cycles of cohesin binding and unbinding to the DNA result in a loop that, due to chromatin fluctuation, increases in size over time. Experimental data have verified predictions from theoretical simulations of the model and fit comfortably with the chromosome properties of yeast genomes. The diffusion capture model was initially developed to explain how cohesin-related SMC complex condensin transforms interphase chromatin into mitotic chromosomes. However, it can help to explain the mechanism of other SMC complexes, including cohesin. The model is applicable for a single cohesin containing dual static and adynamic DNA binding sites or a dimer in which the opening and closing of each cohesin monomer are coordinated.

## The sister chromatid cohesion mechanism

A fundamental property of cells is the equal segregation of genetic material to daughter cells. This is achieved by enforcing the attachment of sister chromatids to spindle fibers in opposite directions. Physical tethering of sister chromatids mediated by cohesin complexes, especially in the centromere region, opposes the pulling force induced by the spindle (Figure 1C). This equilibrium of forces is the molecular basis upon which the chromatids’ attachment to the spindle microtubules in opposite directions is assured. Centromeres are specialized chromatin regions that adopt an elongated shape composed of a dense array of short loops organized around a central backbone. Cohesin, heavily enriched at pericentromeric regions, is essential for the proper organization of the region that is required for supporting kinetochore binding and generating the opposing spindle force (Lawrimore et al., 2015; Lawrimore et al., 2017; Lawrimore and Bloom, 2019; Paldi et al., 2020; Lawrimore and Bloom, 2022). Cohesin removal from the centromeres at anaphase onset relieves the tethering force, and the sister chromatids are pulled to the opposing poles of the dividing cell (Figure 1C) (Michaelis et al., 1997; Megee et al., 1999; Onn et al., 2008). In addition to centromeres, cohesin is resident in many loci along chromosomal arms, called cohesin-associated regions. However, unlike the massive enrichment of cohesin in at pericentromers, these regions comprise only 3–20 cohesins per site (Ghaemmaghami et al., 2003; Weitzer et al., 2003; Glynn et al., 2004; Onn and Koshland, 2011; Gu et al., 2020).

Cohesin is loaded onto the DNA at mitosis exit in mammalian cells or the late G1 phase of the cell cycle in yeast. Loading is mediated by physical interaction with the Scc2 loader at nucleosome-free regions on the DNA (Ciosk et al., 2000; Takahashi et al., 2004). During the S-phase, in coordination with the passage of the replication fork, cohesin captures newly synthesized DNA. Single-stranded DNA regions characterized formed during the lagging-strand synthesis have been shown to enhance the capture of the newly formed DNA strand. Soon after the passage of the replication fork and the entrapment of the formed sister chromatids by cohesin, Smc3 is acetylated on two adjacent lysines located in the head by Eco1 (ESCO in mammalian cells) (Zhang et al., 2008a; Rolef Ben-Shahar et al., 2008; Chao et al., 2017). It has been suggested that acetylation suppresses the ATPase activity of the SMCs and locks cohesin into a pro-tethering conformation (Matityahu and Onn, 2021). Cohesion is maintained during the G2 phase of the cell cycle as Pds5 protects Scc1 from ubiquitination and degradation (D'Ambrosio and Lavoie, 2014; Hartman et al., 2000; Panizza et al., 2000). When cells enter mitosis, cohesin is gradually released and removed from chromosomes. It first dissociates from chromosomal arms and, thereafter, from centromeres *via* proteolysis of Scc1 (Waizenegger et al., 2000; Hauf et al., 2001). Different models of chromatid tethering by cohesin have been suggested (Huang et al., 2005). Notably, one of the main differences between the various models is how many cohesin complexes are required for tethering, a cohesin monomer, a dimer or an oligomer.

## Cohesin stoichiometry

The simplest concept that emerged to explain cohesion is called the embrace or the ring model, which asserts that a single complex entraps two distinct chromatin fibers and tethers them. Early reports suggested that the two chromatids are topologically entrapped in the lumen formed by the Smc1 and Smc3 proteins (Campbell and Cohen-Fix, 2002; Nasmyth and Schleiffer, 2004). Eventually, it became clear that this straightforward model was insufficient, and more complex models followed (Bauer et al., 2021). However, these posed no challenge to the basic view of the cohesin monomer as the active unit. Regardless, an opposing view developed not long after the ring model was proposed (Huang et al., 2005). The snap, bracelet, and handcuff models suggested that each cohesin complex entraps a single chromatid, with cohesion being the result of the dimerization of two cohesins. Conceptual models for loop extrusion and cohesin mediated by a monomer or a dimer are described in Figures 1B,C. Due to these conflicting opinions, many studies and various experimental approaches ensued to determine the stoichiometry of cohesin.

## Cohesin as monomer

**FIGURE 2:**
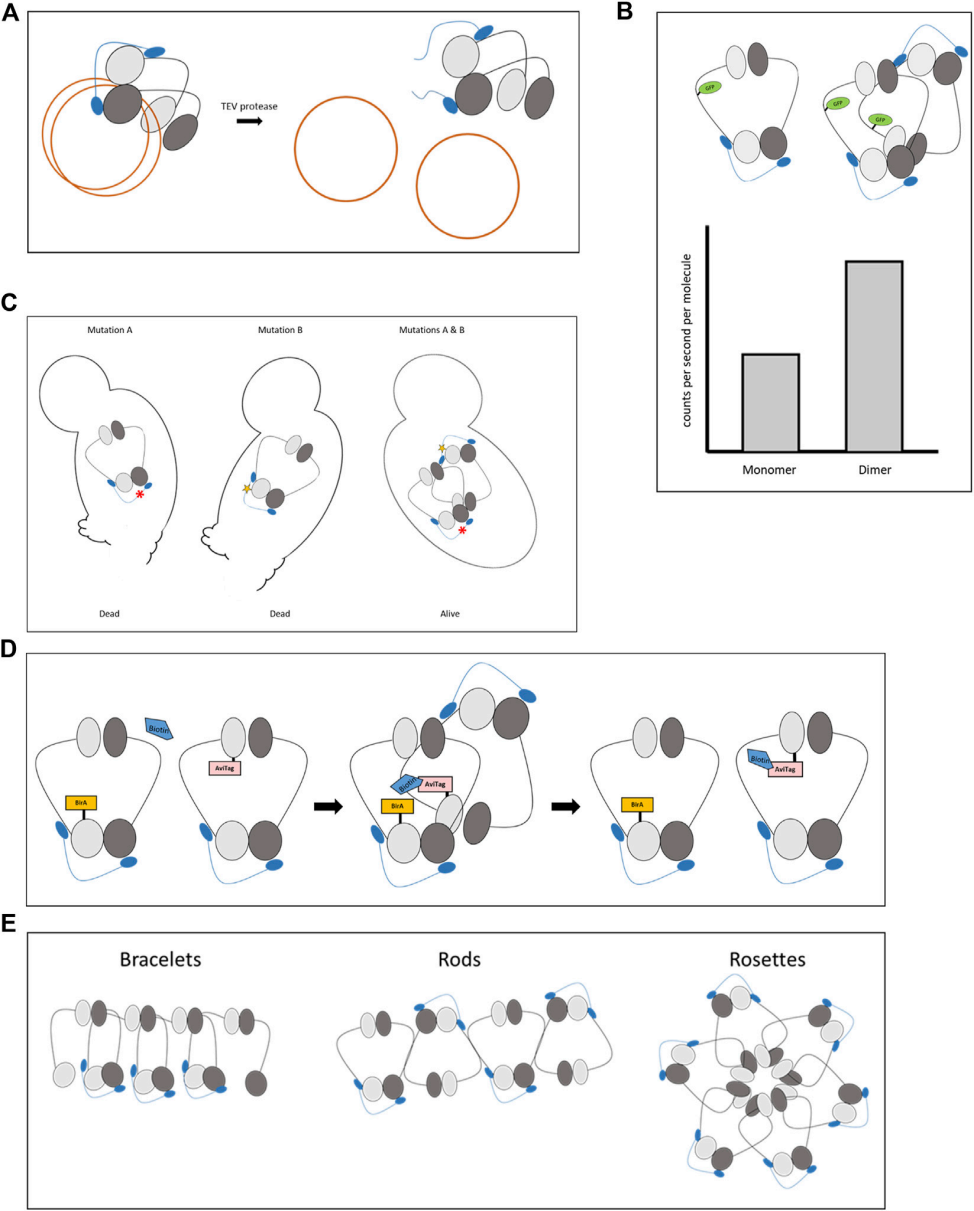
Methods used to determine cohesin stoichiometry. **(A)**. Minichromosome cohesion assay. The minichromosomes are purified and their tethering is determined by a biochemical assay. **(B)**. Photon counting histogram (PCH). The number of photons emitted from a single molecule is counted. Interaction between two proteins will double the amount of the emitted photons in a given time. **(C)**. Interallelic complementation. Cell viability (or any other phenotype) is restored in the presence of two alleles with two mutant alleles that cause inviability when present as a sole copy in the cell. Cells containing both alleles are viable if the encoded mutant proteins interact and the biological activity is restored. **(D)**. BirA proximity biotinylation of AviTag. A protein fused to bacterial BirA biotinylates nearby proteins fused to the AviTag sequence. The biotin remains as a marker for the encounter even if the interacting proteins dissociate. **(E)**. Possible configurations of cohesin clustering: bracelets, rods, and rosettes.

The first convincing evidence that cohesin tethers chromatids as a monomer came from analyzing the entrapment of minichromosomes by cohesin. Yeast cells containing minichromosomes were grown, and the minichromosomes were affinity purified from the cells. Separation of the minichromosomes by sucrose-gradient centrifugation allowed for a differentiation between non-cohesed and cohesed subpopulations (Figure 2A) (Ivanov and Nasmyth, 2005; Ivanov and Nasmyth, 2007). Cohesed minichromosomes appeared in S-phase and were dependent on the activity of cohesin and Eco1. In contrast, minichromosome cohesion was lost when cohesin was cleaved and removed from the DNA *via* site-specific proteolysis by TEV protease (Figure 2A). This work was followed by another study using the same experimental setup with a version of cohesin in which key residues were replaced with cysteines. This modification allowed crosslinking of neighboring subunits. By estimating the efficiency of the crosslinking that fits with the theoretical calculation of a monomer but not a dimer, this investigation determined that cohesion is mediated by a cohesin monomer (Haering et al., 2008).

Spectroscopic studies of photon counting histogram (PCH) supported biochemical evidence of cohesin monomers being the active units (Liu et al., 2020). PCH is a high-resolution technique in which the photons emitted from a single molecule at a nanosecond scale are counted. Comparing the counted photon to a standard curve allows for a determination of the stoichiometry of the emitting proteins. A dimer will emit twice the number of photons per second than the corresponding monomer (Figure 2B). Cohesin subunits were fused to a green fluorescent protein (GFP) in yeast. PCH analysis revealed that cohesin is a monomer throughout the cell cycle, from late G1/S phases when the complex is assembled until its removal from the chromosomes in anaphase. Depletion of key regulators, including the Scc2 loader, Pds5, or Eco1 did not change the monomeric stage of the complex. As a control, two different cohesin subunits were fused to GFP. Under this condition, PCH revealed twice the amount of photons than those emitted from complexes carrying a single GFP, suggesting that detection of cohesin dimerization, if it occurs, was feasible.

The relationship between cohesin tethering and loop extrusion has not been fully established. However, it is reasonable to assume that the stoichiometry of cohesin in both processes is identical. Recently, experimental systems to study loop extrusion by cohesin in real-time have been described (Davidson et al., 2019; Kim et al., 2019; Xiang and Koshland, 2021). Recombinant cohesin is added to DNA attached to a chip, and the extrusion reaction is visualized in real-time. One of the measured parameters in these experiments is the number of extruding complexes. In most of the experiments, cohesin was mediated by a single complex, supporting the concept that monomeric cohesin is sufficient for the loop extrusion activity, similar to the requirement for tethering. However, in some experiments, cohesin oligomers ranging from 2 to more than 7 complexes were detected, suggesting that cohesin oligomerization is possible and can occur under certain conditions (Davidson et al., 2019; Kim et al., 2019; Xiang and Koshland, 2021). Notably, multiple complexes seem not essential for the activity but might have other biological significance.

## Cohesin functions as a dimer (or an oligomer)

Soon after the cohesin monomer embrace model was proposed, it was challenged by the competing bracelet, snap, and handcuff models, which suggested that cohesion is mediated by an oligomer of two or more cohesin complexes that interact with one another (Huang et al., 2005). However, in contrast to early biochemical studies supporting the idea that the cohesin monomer is the active unit, experimental evidence supporting theoretical models of dimerization had been lacking for some time.

Transition electron microscopy analysis of minichromosomes purified from M-phase yeast cells revealed rod-shaped structures at or near the junction between two tethered minichromosomes (Surcel et al., 2008). With a high degree of confidence, these rods were identified as cohesin. Measurements of the geometrical dimensions of the rods led to the idea that cohesin form oligomers through the interaction of the SMC proteins coiled coils leading to the formation of a higher-order tetrameric coil.

The first evidence came from a work supporting the handcuff model in which two cohesin tripartite Smc1-Smc3-Rad21 rings interact through a single Scc3/STAG subunit (Zhang et al., 2008b; Zhang and Pati, 2009). The proof of this model came from a series of experiments conducted in a human cell line that included co-immunoprecipitation, a yeast two-hybrid system, and Förster resonance energy transfer (FRET). Mutations in the SA-binding motif in STAG reduced Rad21-Rad21 interaction (Zhang et al., 2013). Although these studies used multiple methods to demonstrate cohesin dimerization through STAG, the results were not recapitulated by experiments in other organisms until recently, a study in yeast revealed that yeast Rad21 and STAG homologs, Scc1 and Scc3, respectively, are involved in cohesin clustering (Xiang and Koshland, 2021). This latter study is further discussed below.

Another indirect support for cohesin dimerization came from the finding that Eco1 is a dimer in yeast (Onn et al., 2009; Chao et al., 2017). The biological importance of this can be explained if Eco1 acetylates two cohesins simultaneously to enhance their interaction. The idea of cohesin dimerization was strengthened further by genetic studies of interallelic complementation (Figure 2C). In these experiments, mutations in two cohesin subunits were introduced. While the presence of either mutation in cells was not sufficient to support cohesin activity, introducing the two different mutant alleles into one cell restored cohesin activity. Interallelic complementations were detected between the allele combination of Mcd1 as well as Smc3 mutants (Eng et al., 2014; Robison et al., 2018). Surprisingly, the cells containing these allele pairs were viable and displayed wild-type cohesion levels. The interallelic complementation phenotype between alleles of the same subunit implies the formation of a cohesin dimer or oligomer. The mutual presence of the mutated alleles in the same quaternary structure compensates for the loss of function of each mutation. However, these elegant genetic experiments, which provided solid proof of cohesin oligomerization, still lacked molecular evidence.

Molecular confirmation for cohesin-cohesin interaction arrived in a recent study utilizing the proximity biotinylation method (Xiang and Koshland, 2021). In this assay, bacterial BirA recognizes the 15 amino acid AviTag sequence and adds biotin to a lysine residue within the tag. When BirA and AviTag are fused to different proteins, biotinylation will occur only if the proteins are in close proximity and the covalently attached biotin remains as a marker for the encounter (Figure 2D). BirA and AviTag were fused to different cohesin subunits, and the formation of cohesin clusters was recorded in a cell cycle-dependent manner. The clusters were ordered such that the head domain of one cohesin molecule was placed near the head and hinge domains of the other cohesin molecules of the same oligomer. The clusters associated with cohesin enrichment regions on the chromosomes appeared and peaked in the S phase of the cell cycle and partially dissolved from the G2 to the M phase. Cohesin clustering was mediated by the cohesin regulatory subunit Pds5.

The formation of cohesin clusters on chromosomes might have been indisputable proof of the link between cohesin multimerization and function. However, other puzzling results showed that neither cohesin loading by Scc2, nor its acetylation by Eco1 is essential for cohesin clustering that can occur on or off chromosomes. Furthermore, the formation of cohesin clusters *per se* is insufficient to promote its function in cells. As mentioned above, cohesin clusters of 2-7 monomers have been shown to cooperate in loop extrusion (Kim et al., 2019). The presence of one or two cohesins regulates uni-vs. bidirectional extrusion. Therefore, while cohesin monomers are the minimum working unit in both tethering and loop extrusion, dimerization and oligomerization occur on and off chromosomes. Nonetheless, the regulation of cohesin interaction and its functional importance are not yet fully understood.

Spherical clusters of yeast cohesin were formed spontaneously *in vitro* in an ATP-independent manner when cohesin monomers were mixed with DNA. In contrast to the clusters identified in cells, these clusters of 170–1,200 cohesins were formed only on DNA. Loosely compacted DNA loops were detected in the outer periphery of the body. Several properties suggest that these cohesin-DNA bodies are liquid droplets. Their assembly kinetics, merging of neighboring clusters into a single spherical body, fast turnover of cohesin between the droplet and the environment, and reversibility of their formation. These properties imply that cohesin clustering serves as a type of phase separation (Ryu et al., 2021).

## Open questions and concluding remarks

Resolution of the long-standing depute about whether the cohesin functional unit is a monomer, a dimer, or an oligomer is nearing, but the new data raises many new questions. Biochemical, molecular, and imaging studies conclusively show that cohesin monomers are sufficient for both loop extrusion and sister chromatid tethering. However, convincing data that cohesin monomers cluster in a cell cycle-dependent manner suggests a complicated reality.

Above all else: What is the biological significance of cohesin clustering? It has been suggested that clustering is a higher-order organization of cohesin that induces phase separation, a process that leads to the formation of dense chromatin and diluted nucleoplasm phases. In short, phase separation can strengthen tethered regions held by cohesin monomers (Xiang and Koshland, 2021). This property may be particularly important in centromeres where cohesion is essential to ensure their bipolar attachment to the spindle and oppose the pulling forces until anaphase onset. In addition, phase separation can explain how cohesin stabilizes DNA loops (Ryu et al., 2021). However, this model still leaves enigmatic the importance of off-chromosome cohesin clusters, which may serve as an emergency pool in cellular crisis. For example, in response to a double-strand DNA break, cohesin is loaded next to the break site but also genome-wide (Unal et al., 2007). The off-chromosome clusters can rapidly transform into active monomers under such conditions to satisfy the immediate cellular need for active cohesin.

Several mechanisms may affect the shift between the monomeric and oligomeric states of cohesin. The chromosomal localization of cohesin may play a critical role in its tendency to oligomerize. Pericentromeric regions are heavily enriched with cohesin, which is essential for the proper structural organization of the region (Lawrimore et al., 2015; Lawrimore et al., 2017; Lawrimore and Bloom, 2019; He et al., 2020; Lawrimore and Bloom, 2022). The high cohesin density can be a critical factor in enhancing oligomerization. On chromosomal arms, cohesin is less abounded and lesser important for ensuring proper segregation (Ghaemmaghami et al., 2003; Weitzer et al., 2003; Glynn et al., 2004; Onn and Koshland, 2011). Thus, cohesin function as a monomer or individual dimers may be sufficient for roles such as restricting the free diffusion of chromatin, stabilizing loops and regulating transcription. In this context, setting transcription start site (TSS) architecture by RNA polymerase and enhancers unveiled as cohesin recruitment factors for these loci (Zhang et al., 2021; Rinzema et al., 2022). Cohesin clustering at chromosomal arms may occur when loaded near sites of double-strand DNA breaks to strengthen tethering.

The subunit composition of cohesin is another mechanism that may affect its ability to oligomerize. Higher eukaryotic cells encode for several alternative cohesin subunits, which can give rise to alternative complexes (e.g., STAG1/STAG2 and PDS5A/PDS5B) (Onn et al., 2008). Some subunits may enhance oligomerization, while a complex containing the paralog subunit will remain a monomer.

Finally, post-translational modifications can control the shift of cohesin from monomeric to oligomeric form. Specifically, Smc3 acetylation by Eco1, which is critical for cohesion establishment, does not affect oligomerization. However, other modifiers may enhance the inter-cohesin interactions. A future challenge is identifying factors that control cohesin oligomerization and dissecting the relationship between cohesin complex stoichiometry and biological function.

Pds5 is the factor that controls cohesin clustering (Xiang and Koshland, 2021). Previously, this protein has been shown to protect Mcd1 from ubiquitination and degradation post-cohesion establishment, and thus, it plays a role in cohesin maintenance during G2 (Hartman et al., 2000; Panizza et al., 2000). Pds5 is essential for cell viability, and in its absence, cohesion is lost. A new yeast study showed that cells can live without Pds5 under one of two conditions - overexpression of Mcd1 or an accumulation of the loading clamp PCNA on chromosomes (Choudhary et al., 2022). These modifications stabilize cohesin on chromatin. It would be interesting to test how these affect cohesin clustering and what other phenotypes are associated with clustering loss. For example, are these cells sensitive to DNA damage?

It is easier to imagine the role of cohesin dimerization in loop extrusion activity. Cohesin stoichiometry can control uni or bidirectional extrusion. Indeed, both activities have been detected. However, it is not clear if there is a biological difference between them. Is it possible that while genome organization is achieved rapidly by bidirectional extrusion, mild changes related to internal or environmental signals are mediated by a cohesin monomer performing unidirectional extrusion?

The exact configuration of cohesin clusters has yet to be determined. However, several experiments have provided clues for cluster organization, and numerous models have been described (Huang et al., 2005; Guillou et al., 2010; Stephens et al., 2013; Skibbens, 2016). In short, the three popular conformations are bracelets, rods, and rosettes (Figure 2E). Bracelets are formed by intercomplex dimerization of the Smc1 and Smc3 heads. Rods are created through the interaction of the coiled coils. Ordered interaction of the heads or hinges lead to Rosettes. Importantly, one must consider the possibility that these models are not exclusive of one another and that organization details may vary depending on chromatin structure and regulators.

Advances in two objectives are needed to further clarify the importance of cohesin stoichiometry. The first is to comprehensively understand all of the molecular events involved in both loop extrusion and tethering activities. The second is to dissect in detail the basis for oligomerization to gain full knowledge of the mechanism, its regulation, and biological significance. The cohesin numbers game is not over.
